# An Elastic Fine-Tuning Dual Recurrent Framework for Non-Rigid Point Cloud Registration

**DOI:** 10.3390/s25113525

**Published:** 2025-06-03

**Authors:** Munan Yuan, Xiru Li, Haibao Tan

**Affiliations:** 1 Hefei Institute of Physical Science, Chinese Academy of Sciences, Hefei 230031, China; mnyuan@hfcas.ac.cn (M.Y.); hbtan@hfcas.ac.cn (H.T.); 2 Zhongke Technology Achievement Transfer and Transformation Center of Henan Province, Zhengzhou 450046, China

**Keywords:** non-rigid registration, 3D modeling, unsupervised, elastic fine-tuning, dual recurrent computation

## Abstract

Non-rigid transformation is based on rigid transformation by adding distortions to form a more complex but more consistent common scene. Many advanced non-rigid alignment models are implemented using supervised learning; however, the large number of labels required for the training process makes their application difficult. Here, an elastic fine-tuning dual recurrent computation for unsupervised non-rigid registration is proposed. At first, we transform a non-rigid transformation into a series of combinations of rigid transformations using an outer recurrent computational network. Then, the inner loop layer computes elastic-controlled rigid incremental transformations by controlling the threshold to obtain a finely coherent rigid transformation. Finally, we design and implement loss functions that constrain deformations and keep transformations as rigid as possible. Extensive experiments validate that the proposed method achieves state-of-the-art performance with 0.01219 earth mover’s distances (EMDs) and 0.0153 root mean square error (RMSE) in non-rigid and rigid scenes, respectively.

## 1. Introduction

Motivated by the development of the meta-universe industry, 3D models demonstrate enhanced applicability in small-scale indoor entertainment environments, such as indoor outfit change. The 3D point cloud obtained through consumer-level depth sensing devices tends to become the main carrier of information exchange between the real world and parallel virtual worlds [1,2,3,4]. The small-scale indoor 3D point data manifests two characteristics: (1) the low-cost depth-sensing device usually obtains partial geometric representations due to constrained viewpoint sampling; (2) the short-range measurement configuration captures detailed geometric features and demonstrates sensitivity to morphological deformations. Non-rigid registration technology emilites point cloud global transformations and local distortions to align source and target objects, which is the fundamental step of 3D data application. Although much pioneering research of point cloud registration has been proposed, the non-rigid registration problem is still a difficult problem that many researchers continue to study [5,6,7,8].

In many traditional optimization-based studies, non-rigid registration usually converts shape distortions’ (may differ by bending and stretching) alignment to minimize the distance between surfaces with regular expressions, such as Tukey, Welsch, and Geman–McCLure, which corrects the registration process in terms of smoothness, point location, local topology, etc. [9,10,11]. However, the discrete nature of the point cloud prevents the use of micro-miniaturized mathematical properties in computation. Furthermore, many studies have transformed the non-rigid alignment problem into a nonlinear and non-convex mathematical optimization problem. As a representative, coherent point drift (CPD) based on optimal probability distribution is the most widely used non-rigid registration method [12]. Registration based on iterative computation has the problems of low convergence speed and a poor smoothing effect, which makes the non-rigid alignment focus on solving the dense real correspondence point relationship [13,14,15]. This model-driven point correspondence estimation is obtained by calculating the correlation between manual feature points and descriptors, which is not deformation-resistant and is easily affected by noise, density, and overlap rate variations [11,16,17,18].

In recent years, the learning-based framework has demonstrated the capability to establish data-driven point cloud correspondences through feature-based matching or shape-matching techniques [5,19,20,21,22]. The problems of these methods conclude that a large number of labeled data are required during training processes or predetermined internal structures are set, such as lattice representations, in the input data, which significantly increases the difficulty of acquiring training datasets and restricts broader applications. Currently, many non-rigid registration models based on unsupervised learning have been proposed and demonstrated state-of-the-art performance [6,23,24,25,26]. However, existing similarity-constrained loss functions have substantial deficiencies in effectively driving the network to learn optimal solutions.

To address these challenges, this study investigates unsupervised learning for non-rigid registration by leveraging complementary advantages of feature and shape matching while considering the characteristics of small-scale scene point clouds. Combining the recurrent neural network representation of the LK algorithm, we propose a dual recurrent registration framework, NGRLK, that decomposes non-rigid transformations into a series of elastic fine-tuning rigid transforms. To be specific, based on the computation process with iteratively solving rigid transform increments, the point pairs’ correspondence can be continuously computed and updated. Through updating the weights of rigid transformation through an attention mechanism to eliminate the influence of invalid transformations, the latest transformed source point cloud is concatenated from the combination of rigid transformation with corresponding weight in each round, which is fed into another recurrent neural network to output the aligned results in an end-to-end manner. Moreover, to tackle the shape similarity metric problem, we further design a loss function of local spatial consistency measure to constrain deformation similarity, which is complementary to the two-dimensional manifold representation of the point cloud for deformation shape similarity measurement. To the best of our knowledge, extensive experiments have validated that the NGRLK proposed in this paper has advanced performance compared with other SOTA non-rigid registration methods on both synthetic and real scanning datasets.

Thus, the main contributions of this paper are as follows:

(1) We present that a point cloud non-rigid transform can comprise some rigid transforms with different weights.
(2) We achieve fine-tuning incremental changes between successive rigid transformations, which increases the coherence and edge smoothness from rigid to non-rigid transformations.
(3) We propose a local spatial consistency metric loss function to compute similarities between the transformed neighborhoods of corresponding points, which makes the rigid incremental transformations sufficiently small.
(4) Extensive experiments have been conducted on various datasets for the point cloud non-rigid and rigid registration, which shows that the method proposed in this paper has SOTA performance.

## 2. Related Work

Rigid registration based on the PointNet framework. As a milestone in point cloud feature learning, PointNet is the first end-to-end point cloud solution that can directly perform feature learning on points [27]. Many excellent point cloud feature extraction methods based on the PointNet framework have been proposed. To improve the expression abilities of the point features extracted from the PointNet, the method mentioned in [28] treats the PointNet features as global features that are joined with local features. For the problem of information loss of maximum pooling in PointNet, PointNetX conducts compensation by stratifying and fusing global features from different layers [29]. Specifically for point cloud registration, inspired by PointNet, PPFNet embeds global information with local feature descriptors to enhance the expressiveness of features, which facilitates matching accuracy [30]. PPF-FoldNet utilizes PointNet as an encoder–decoder for point features and optimizes the learning model based on the Chamfer loss function to obtain correspondences [31]. PointNetLK utilizes PointNet to extract point cloud data global features and computes the transformation matrix with an inverse synthesis algorithm [32]. PCRNet [33], inspired by PointNetLK, employs PointNet to encode location and shape information into feature vectors for alignment, which effectively improves the robustness to noise. Inspired by these methods, we have designed an incrementally controllable rigid transformation estimation based on the recurrent neural network representation of the LK algorithm. Different from previous studies, our approach is not only applicable to rigid registration but can also be extended to non-rigid registration.

Non-rigid registration based on supervised learning. Different from registration models with iterative optimization computation affected by variations in noise, density, and overlap to degrade performance, the convolutional neural network deeply extracts point information to obtain distinctive feature expressions, which determines highly accurate correspondence [5]. By learning the surface representation of discrete surfaces, DiffusionNet computes the correspondence between a pair of shapes to accomplish non-rigid transformation [34]. For the DispVoxNets, the non-rigid registration result can be directly obtained through the learning of displacement fields and parameter representations. By performing a voxel mesh representation of point clouds, the convolutional neural network is utilized to compute the voxel displacement field to accomplish overall and refine deformation [35]. The SMPL model (Skinned Multi-Person Linear Model) is widely used to characterize human morphology, and the model parameters are continuously trained and optimized for the non-rigid registration of human 3D morphology [20,21,22]. Among many learning-based methods, the correspondence performance with the transform mechanism stands out. Trappolini et al. [36] designed a surface attention mechanism to obtain point correspondences, which was insensitive to point cloud density. Boscaini et al. [37] trained the high-accuracy shape feature correspondences by anisotropy fused with a convolutional neural network in different weights. To improve the correspondence accuracy with missing data, Attaiki et al. [19] integrated the shape features with a cross-attention module, which assigned the non-overlapping features a lower weight to preserve the overlapping regions as much as possible. All these supervised learning methods minimize errors and require labeled correspondence, which is usually unavailable for registration tasks. Incorporating shape feature correspondences with attention mechanisms, this paper focuses on non-rigid registration estimation through unsupervised learning.

Non-rigid registration based on unsupervised learning. Through registration error constraints, such as Gaussian loss and Chamfer distance, in conjunction with optimization regularity terms, many non-rigid registration methods that directly model the data have emerged. The PR-Net models the point cloud to be aligned based on thin plate spline templates, which utilize mesh assistance for shape feature learning and constrain the alignment accuracy by GMM losses [23]. The CPD-Net learns geometric transformations of varying complexity through a neural network and uses Chamfer distances to constrain the alignment direction, which is appropriate for scenarios with anomalous and incomplete point correspondences [24]. Halimi et al. [25] designed an unsupervised learning method for approximating the preservation of surface metric structures due to deformations, which conducted non-rigid registration by constraining the minimum deformation correspondence. The RMA-Net designs a continuously differentiable end-to-end cyclic framework to iteratively solve small rigid transformations and approximates global non-rigid transformations by solving multi-view projected shape similarity errors [6]. The CorrNet3D designs and implements a symmetric deformer in conjunction with a 2D flow shape and point correspondence, which constrains the learning process with point-to-point loss [7]. The GP-Aligner designs a model-free descriptor to characterize shape correlations among groups of point sets, which implements a novel unsupervised method [38]. Currently, unsupervised non-rigid registration methods have demonstrated state-of-the-art (SOTA) performance. However, most existing methods predominantly depend on per-point features or shape constraints. In contrast, the approach proposed in this paper integrates the complementary strengths of feature matching and shape constraints, without any shape templates, achieving a dual recurrent non-rigid registration framework. Furthermore, a novel loss function is designed to enforce spatial similarity constraints.

## 3. Problem Formulation

**Figure 1:**
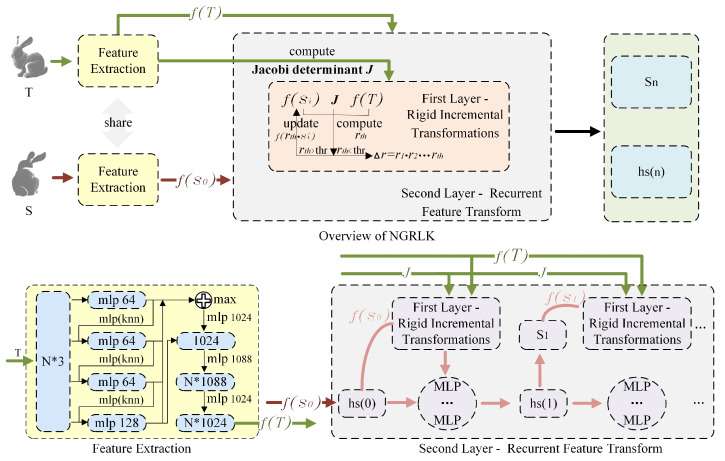
The main framework of NGRLK.

Inspired by the RMANet implementation, we approximately convert the non-rigid transforms into a series of rigid transforms of different weights by recurrent neural networks. Moreover, we incorporate the recurrent rigid transformation computation similar to PointNetLK to obtain rigid incremental transformations. Ultimately, we convert the non-rigid alignment process into a two-layer recurrent point cloud feature transform fitting computation and propose our non-rigid registration framework NGRLK, as shown in Figure 1.

Let f denote the point feature extraction $R^{N\times 3}\to R^{\left(N+1\right)\times K}$ and N be the point number. The N K-dimensional vectors embedded with global and local features are computed with f for a point cloud set. When two point clouds are closer to each other, the features are more similar. The point cloud registration problem can be converted into solving the transformation of point cloud features, and the non-rigid point cloud registration can be defined in Equation (1):

(1) $$f\left(T\right)\approx f\left[\left(\prod △r\right)\cdot S\right]$$

where T denotes the target point cloud, S is the source point cloud, and △r represents a rigid transformation.

Based on the derivation of the Inverse Compositional (IC) formulation in reference [39], Equation (1) can be transformed into Equation (2):

(2) $$f\left(S\right)\approx f\left(T\right)+\frac{\partial }{\partial \varepsilon }\left[f\left({△r}^{-1}\cdot t\right)\right]\varepsilon$$

where t is an exponential mapping containing the distortion parameter ε and $\frac{\partial }{\partial \varepsilon }\left[f\left({△r}^{-1}\cdot t\right)\right]$ denotes the Jacobi determinant *J*.

The rigid incremental transformation r is obtained by the LK algorithm in pointnetLK [32] for each column in J and expressed as Equation (3):

(3) $$\begin{array}{c} J_{col}=\frac{f\left(\exp\left(-∆\varepsilon t\right)\cdot T\right)-f\left(T\right)}{∆\varepsilon }\\ \varepsilon =J^{+}\left[f\left(S\right)-f\left(T\right)\right]\\ r^{-1}=\exp\left(-\sum_{i}\varepsilon_{i}t\right) \end{array}$$

where ∆ε is the infinitesimal perturbation of ε and usually empirically set as some small constant value over all iterations, $J^{+}$ is the Moore–Penrose generalized inverse of the Jacobi determinant, and i is the number of columns in J.

For the approximate equality sign ≈ instead of the equality sign = in Equations (1) and (2), the above equations express a single rigid accumulation transformation. For each new rigid transformation $△r=r_{1}\cdot r_{2}\cdot r_{3}\cdot \ldots r_{th}$, the number of cycles th is determined by adjusting the threshold value and able to control the amplitude of the rigid incremental transformation, and the corresponding rigidity cumulative transformations R=∏△r should be transformed. For $f\left[\left(\prod △r\right)\cdot S\right]$, S is the most recently transformed source point cloud instead of the initial source point cloud. Through the attention mechanism, each transformed source point cloud is assigned a weighted value ωS, and the non-rigid transform should be expressed as an accumulation of transformed source point clouds with different weights, as shown in Equation (4):

(4) $$f\left(T\right)=\sum_{i=1}^{k}w_{i}f_{i}\left[\left(\prod △r\right)\cdot S\right]$$

where the weights w should satisfy the restriction $\sum_{i=1}^{k}w_{i}=1$ to eliminate jumps like the joints of structural components.

## 4. Methodology

The non-rigid registration modeling framework NGRLK proposed in this paper mainly contains feature extraction, approximation transformation module, and loss function constraints modules. The key to this achievement is the combination of point cloud feature computation, point cloud feature correspondence estimation, elastic rigid transformation computation, and non-rigid transformation constraints. Although the outer-layer recurrent rigid computation framework is similar to the RMANet, the rigid transformation is elastically controllable by the inner-layer recurrent computation. Moreover, different from PointNetLK, the registration ability of NGRLK has been extended from rigid to non-rigid scenes.

### 4.1. Feature Extraction

The feature acquisition module in the NGRLK is independently pluggable and can be adapted to many feature extraction methods, such as PointNet [27], DGCNN [40], DIRGMR [41], etc. In PointNetLK, the point cloud feature is extracted by the PointNet, which fails to portray localized information and makes the characterization incomplete. As an improvement, we utilize an advanced feature extraction method published by DGCNN, which represents the point cloud as a graph structure and considers the topology of neighboring points to form a strong representation. The target point cloud features are used to compute the Jacobi J and jointly performed with the source point cloud features to obtain the similarity estimation, which can be transformed into a point correspondence matrix. In the whole process, the feature extraction of the target point cloud only needs to be performed once, and the features of the source point cloud and the point correspondence estimation between the two point cloud sets need to be computed several times.

### 4.2. Approximation Transformation Module

**Figure 2:**
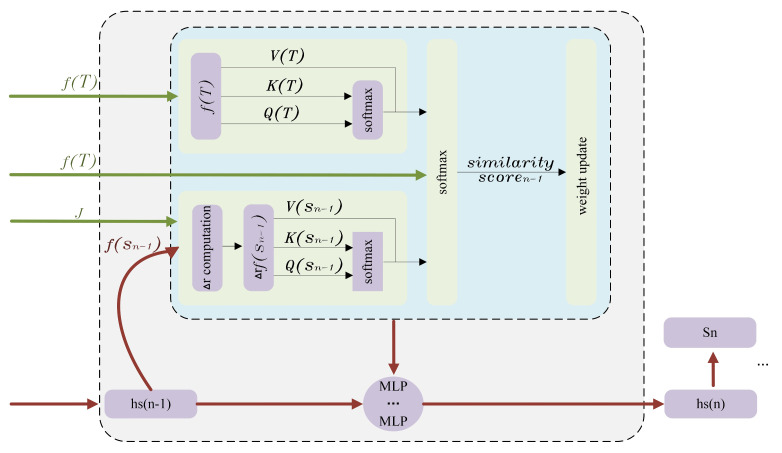
The computation and update of each unit.

The key implementation of NGRLK lies in the double-loop computation of the fine rigid transformation, which is superimposed to form the final non-rigid transformation. First, the NGRLK simulates the overall process of approximating rigid transformation to non-rigid transformation through the first outer iterative computation, which is essentially a recurrent neural network. Inspired by the implementation of RAFT and RMA-NET [6], the NGRLK learns the weight coefficients of △r changes by utilizing a gated loop unit GRU update incorporated with an attention mechanism. From the initial state of the GRU unit, each iterative computation generates an update direction. The transformed features, correlations, and hidden states serve as inputs to the GRU, which outputs the updated hidden state and the rigid transformation. Before performing the first computation, the feature $f\left(S_{0}\right)$ extracted from DGCNN and zero-initialized hidden state $hs\left(0\right)$ are employed as the initial feature and hidden state to ensure unbiased attention allocation in the model. Furthermore, the adaptive optimizer Adam is adopted to ensure computational convergence and precision. In the n-th computation, the input of GRU contains the hidden state $hs\left(n-1\right)$ and the feature $f\left(S_{n-1}\right)$. Referring to [6], the GRU updates the hidden state as $hs\left(n\right)$. The dense layers in GRU are replaced with MLPs, which update the W list formed by weight recalculation for the n-th target point cloud rigid transformation with the attention mechanism. The magnitude value indicates the influence of the corresponding rigid transformation. Then, referring to the LK algorithm in PointNetLK [32], the NGRLK computes the source point cloud rigid transformation increment r in the second inner layers, which increases the coherence of the transformation by controlling the threshold to change the r computation, as shown in Figure 2.

For the n-th inner-layer computation, let the incrementally transformed source point cloud be denoted as $S^{n}$. At the n-th outer-layer computation, the transformed source point cloud is denoted as $S_{n}$. The incremental transformation follows Equation (5):

(5) $$S^{n}=∆r(r_{1}\cdot r_{2}\cdot r_{3}\cdot \ldots r_{th})S_{n-1}$$

For computational simplicity, this paper sets the threshold high for only one inner-layer incremental rigid transformation computation in each round of outer-layer recurrent computation, and the incremental transformation can be simplified as Equation (6):

(6) $$S^{n}=∆r(r_{1})S_{n-1}$$

Then, the similarity of $f\left(T\right)$ and $f\left(S^{n}\right)$ is compared to obtain the scoring list $similarity\;{score}_{n}$, and the transformation weights are updated according to the normalization of the $similarity\;{score}_{n}$ and composed into a new weight vector $\left(w_{1},w_{2},\ldots ,w_{n}\right)$. The hidden state is updated and the source point cloud S is updated according to Equation (7):

(7) $$S_{n}=w_{1}S^{1}+w_{2}S^{2}+\cdots +w_{n}S^{n}$$

where the normalization of the score list ensures that the accumulative sum of $w_{1}$ to $w_{n}$ is 1. The computation details are presented in Algorithm 1.
**Algorithm 1** Inner-layer computation1**input:** point cloud feature $f\left(T\right)$, point cloud feature $f\left(S_{n-1}\right)$, Jacobi matrix J of T2**output:** the n-th stage point cloud $S_{n}$, weight vector $\left(w_{1},w_{2},\ldots ,w_{n}\right)$3**begin**4      attention compute $f\left(T\right)$ as $f\left(T\right)$’5      compute $r_{i}$ with $f\left(T\right)$, $f\left(S_{n-1}\right)$, and J6**for** $r_{i}$ > threshold value7      update $S^{n}=r_{i}S_{n-1}$8      i=i+1 and compute $r_{i}$ with $f\left(T\right)$, $f\left(S^{n}\right)$, and J9** end for**10      obtain incremental transformation $∆r=r_{1}\cdot r_{2}\cdot r_{3}\cdot \ldots r_{i}$11      compute $f\left(S_{n}\right)$ with incremental transformation ∆r and $f\left(S_{n-1}\right)$12      attention compute $f\left(S_{n}\right)$ as $f\left(S_{n}\right)$’13      update $similarity\;{score}_{n}$ to form new weight vector $\left(w_{1},w_{2},\ldots ,w_{n}\right)$14**end**

### 4.3. Loss Function Constraints

For unsupervised learning, the loss function directly affects the learning results. For the non-rigid change, we can constrain the shape change by taking the chamfer distance (CD) as the loss function, which can be expressed as Equation (8):

(8) $$L_{CD}={\left||S-T|\right|}^{2}+{\left||T-S|\right|}^{2}$$

**Figure 3:**
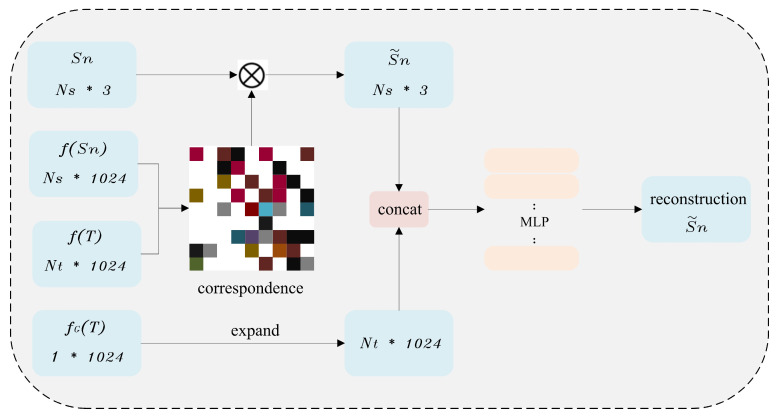
The deformation and reconstruction process from S to T.

However, for severe surface deformation or training overfitting, $L_{CD}$ fails in deforming S to T effectively. During each iterative process, the correspondence matrix between the features of the deformed S and T is recomputed. According to the AtlasNet surface generation method [42], the correspondence matrix can be regarded as a continuous smooth 2D manifold structure, and the multiplication of S with the correspondence matrix, denoted as $\tilde{S}$, can realize the mapping of S to a 2D manifold. Referring to the reconstructed point cloud method used by CorrNet3D [7], the similarity between $f\left(S_{n}\right)$ and $f\left(T\right)$ is computed and the point correspondence can be viewed as continuously smoothed 2D manifolds·. The deformed source point cloud $\tilde{S}$ is obtained by $S_{n}$ cross-multiplying with the correspondence matrix, which preserves the features of $S_{n}$. The $\tilde{S}$ is concatenated with the extension term of $f_{G}\left(T\right)$ to form a combination [$\tilde{S},expand\left(f\left(T\right)\right)$], and the reconstruction from $\tilde{S}$ to the target point cloud $\hat{T}$ is realized by the MLP computation at once, which is an important output of the outer-layer and shown in Figure 3.

According to Equation (9), the difference between T and $\hat{T}$ can be minimized.

(9) $$L_{surf}={\left||T-\hat{T}|\right|}^{2}$$

For the point correspondence matrix *C*, the constraint term expressed in Equation (10) is utilized to minimize the one-to-many point matching.

(10) $$L_{corr}={\left||CC^{T}-I|\right|}^{2}$$

where I denotes the unit matrix.

The spatial distribution of a local point neighborhood can be effectively characterized by a Gaussian model, of which the probability density function can be expressed in Equation (11).

(11) $$f\left(x\right)=\frac{1}{\sqrt{2\pi }\sigma }exp\left(-\frac{{\left(x-\mu \right)}^{2}}{2\sigma^{2}}\right)$$

where μ and $\sigma^{2}$ are denoted as the mean and variance. For the multidimensional data, the Gaussian probability density function (PDF) can be expressed in the form of Equation (12).

(12) $$P\left(x|\theta \right)=\frac{1}{{\left(2\pi \right)}^{\frac{D}{2}}{\left|\sum \right|}^{\frac{1}{2}}}exp\left(-\frac{{\left(X-\mu \right)}^{T}\sum^{-1}\left(X-\mu \right)}{2}\right)$$

where D denotes the dimensionality, μ represents the mean vector across all dimensions, and ∑ signifies the covariance matrix. Based on Equation (12), the 3D point cloud Gaussian distribution can be represented in Equation (13).

(13) $$gauss\left(P\right)=\frac{1}{{\left(2\pi \right)}^{\frac{3}{2}}{\left|\sum (P)\right|}^{\frac{1}{2}}}exp\left(-\psi \left(P\right)\right)$$

where ∑(P) denotes the covariance matrix of point cloud P, $\psi \left(P\right)$ represents $\frac{1}{2}{\left(P_{xyz}-\mu_{P}\right)}^{T}\sum^{-1}\left(P_{xyz}-\mu_{P}\right)$, and $\mu_{P}$ is the component-wise mean values of P. The degree of divergence between probability distributions can be effectively measured by the Kullback–Leibler (KL) divergence. Particularly advantageous is the existence of a closed-form expression for the KL divergence between Gaussian distributions, which substantially simplifies computational implementation. The amount of change in the relative position of the points in local space caused by each transformation of the source point cloud should be sufficiently small. We designed a loss function based on the KL to constrain the spatial distribution of the points as close as possible, as in Equation (14).

(14) $$L_{space}=\sum_{p,q\in E}D_{KL}(p||q)$$

where E is the k-nn neighborhood of the same point (p and q) before and after the incremental transformation of the source point set and $D_{KL}$ denotes the relative entropy of the different distributions. Equation (14) essentially computes the mean log difference between $gauss\left(p\right)$ and $gauss\left(q\right)$ concerning the former, and as the two distributions get closer together, the value of KL becomes smaller, and vice versa the value will keep increasing. For the KL divergence computation in Equation (14), the detailed mathematical derivation can be expanded as shown in Equation (15).

(15) $$D_{KL}(p||q)=\int guass\left(p\right)\left(x\right)log\frac{guass\left(p\right)\left(x\right)}{guass\left(q\right)\left(x\right)}dx$$

Based on Equation (13), the detailed expression of A can be derived as presented in Equation (16).

(16) $$\frac{guass\left(p\right)\left(x\right)}{guass\left(q\right)\left(x\right)}=\frac{\sum_{q}^{1/2}}{\sum_{p}^{1/2}}exp\;(\psi \left(q\right)-\psi \left(p\right))$$

By applying logarithmic operations and evaluating each quadratic term in Equation (15) with the expectation characteristics of Gaussian distributions, the final computation formulation is presented in Equation (17).

(17) $$D_{KL}(p,q)=\frac{1}{2}\left[log\frac{\sum \left(q\right)}{\sum \left(p\right)}+Tr\left({\sum_{q}}^{-1}\sum_{p}\right)-3+\phi \right]$$

where Tr is the matrix trace and ϕ is ${\left(\mu_{q}-\mu_{p}\right)}^{T}\sum \left(q\right)(\mu_{q}-\mu_{p})$.

**Figure 4:**
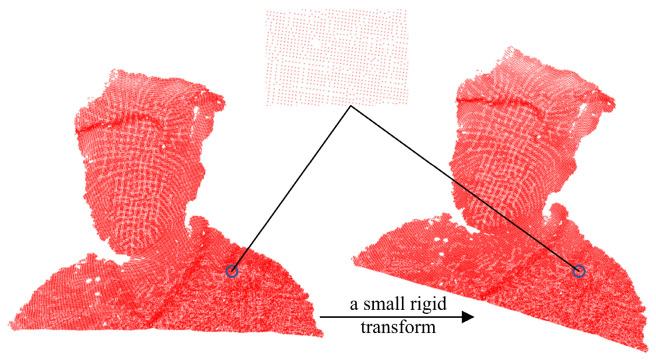
The local distribution of a point in point cloud.

By observing the distribution of points in the neighborhood space before and after the transformation of the point clouds, as shown in Figure 4, this constraint can efficiently keep the change in the local space point cloud distribution small to make the transformation as rigid as possible.

The final loss function is formed as shown in Equation (18):

(18) $$Loss={\lambda_{1}L}_{CD}+L_{surf}+\lambda_{2}L_{corr}+\lambda_{3}L_{space}$$

This section represents the core component of this research. Although an efficient recurrent neural network, such as GRU, is employed, the iterative computation process and loss function evaluation involve quite computation, and efficiency is not an advantage when processing large-scale or complex point clouds. Thanks to the stable sampling environment of small indoor scenes, a uniformly distributed 3D model can usually be obtained. By employing down-sampling algorithms, the computational load can be effectively reduced, making it particularly suitable for non-real-time human–computer interaction experience scenarios.

## 5. Experimentation and Analysis

We implemented the proposed NGRLK model in Python v3.7. The experiment environment is configured as a cluster with the SIMT accelerator made in China. The cluster includes many nodes, each containing one CPU and four accelerators. The CPU has four NUMA nodes, and each NUMA node has 8 × 86-based processors. The accelerator, made in China, adopts a GPU-like architecture consisting of a 16 GB HBM2 device memory and many compute units. For accelerators connected to the CPU with PCI-E, the peak bandwidth of the data transmission between main memory and device memory is 16 GB/s.

### 5.1. Dataset Processing

Non-rigid dataset. Surreal is a standard dataset for large-scale personnel generated from RGB videos, which is 230 K and contains depth, body part, optical flow, 2D/3D human pose, and surface normal information. In this experiment, 115 K datasets are randomly generated for non-rigid alignment training. Moreover, the SHREC is a very challenging dataset for the stronger pose distortion and incomplete shapes, which contains 430 datasets and is only used for non-rigid registration testing. For the two datasets, we use a down-sampling algorithm to select 1024 points as experiment objects.

Rigid dataset. ModelNet40 [43] is an object-level point cloud dataset consisting of 12,311 models from 40 categories. For each category, 80% of models are taken for parameter learning and the rest, 20%, are utilized for validation. For each model, we use a down-sampling algorithm to select 1024 points as experiment objects. Then, the random rotation and translation are performed for each object in the dataset to form the target point cloud datasets, where the rotation is restricted to [0, 45°] and the translation is constrained to [−0.5, 0.5] for X, Y, and Z axes, respectively.

**Figure 5:**
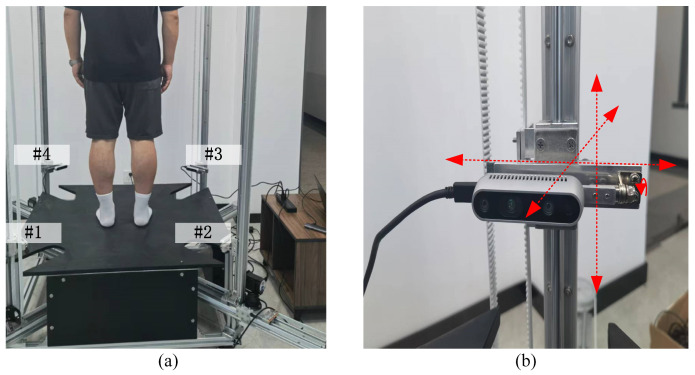
The self-sampled data system. (**a**) The real data sampling platform; (**b**) the design structure sampled camera.

The real sampled data. Self-sampled data is captured from the environment shown in Figure 5 and the red arrows of Figure 5b represent x, y and z axes. The source and target point data are captured from four identical RealSense cameras, which are placed on the four brackets labeled #1, #2, #3, and #4 in Figure 5a for the same scene. To ensure the effective coverage of different-sized objects, the design structure of the camera mount is shown in Figure 5b, which can realize the precise adjustment of the camera in horizontal, vertical, forward, and backward and pitching directions according to different experimental scenes. During the experiment, Hbody01-02 and Fbody01-02 are the sampling data with noise and deformation for non-rigid registration testing, and Face01-02 and Body01-02 are utilized as the representative data for rigid registration validation. The all-sampled data are pre-processed with our previous methodology published in [44].

Since the most complex non-rigid transform object in small indoor scenes is human, the non-rigid experiments focus on human subjects for registration performance. Conversely, the rigid experiments mainly employ object-level targets for evaluation.

### 5.2. Experiment Evaluation

The comparison methods. In order to validate the performance of NGRLK, some methods are selected for comparison in non-rigid and rigid scenes. CPD (2007) [12] is the benchmarking algorithm and is most widely used in the non-rigid registration field. F3D (2019) [45] is a novel deep neural network for non-rigid registration, which directly learns stream embeddings and hierarchical features from point clouds to represent point motion. As a deformation-to-reconstruction non-rigid registration framework, CorrNet3D (2021) [7] is a novel dense correspondence learning model. RMANet (2021) [6] is an advanced non-rigid registration method based on a series of rigid transformations. Lepard (2022) [5] is an excellent model for non-rigid registration based on a deep learning framework to extract point cloud features from space and position perspectives in the last three years. RPM-Net (2020) [46], as an upgraded version of ICP with soft correspondence and simulated annealing, is an excellent rigid registration method. We compare NGRLK to CPD, F3D, CorrNet3D, RMANet, and Lepard on non-rigid correspondence, public datasets, and real sampling data scenes. Moreover, we compare NGRLK to CorrNet3D, RMANet, and the representative rigid registration method RPMNet on public datasets and real sampling data scenes. During the various experiments, these representative registration methods are re-trained. For our NGRLK, the data batch size is 10, the number of loops will be increased from 1 up to 8, the learning rate is 1 × 10^−4^, and the empirical values λ1, λ2, and λ3 of the loss function are set to 0.5.

The evaluation metrics. In the non-rigid experimental session, the overall effect of non-rigid registration is measured by analyzing the Corr (%) defined in CorrNet3D with different error tolerances and the earth mover’s distance (EMD) between the aligned and target datasets for different non-rigid registration algorithms. In the rigid phase, the performance of rigid registration is validated by comparing the Corr (%) and the root mean square error (RMSE) values between the aligned and target objects with different registration algorithms.

### 5.3. Non-Rigid Experiment

**Figure 6:**
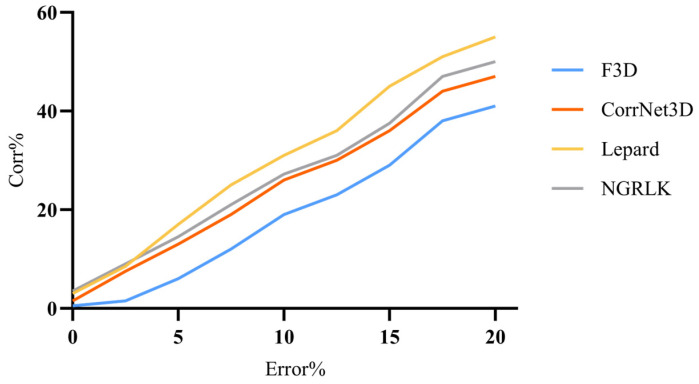
The Corr% of F3D (2019) [45], CorrNet3D (2021) [7], Lepard (2022) [5], and NGRLK.

**Figure 7:**
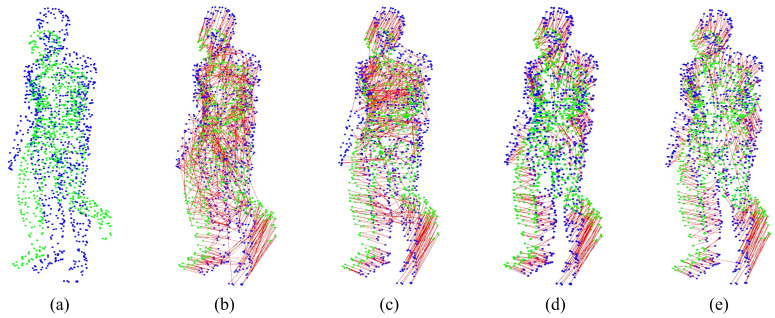
The point correspondences estimation of F3D (2019) [45], CorrNet3D (2021) [7], Lepard (2022) [5], and NGRLK. (**a**) The source point cloud and target point cloud (ground truth); (**b**) the correspondence estimation of F3D; (**c**) the correspondence estimation of CorrNet3D; (**d**) the correspondence estimation of Lepard; (**e**) the correspondence estimation of NGRLK.

**Figure 8:**
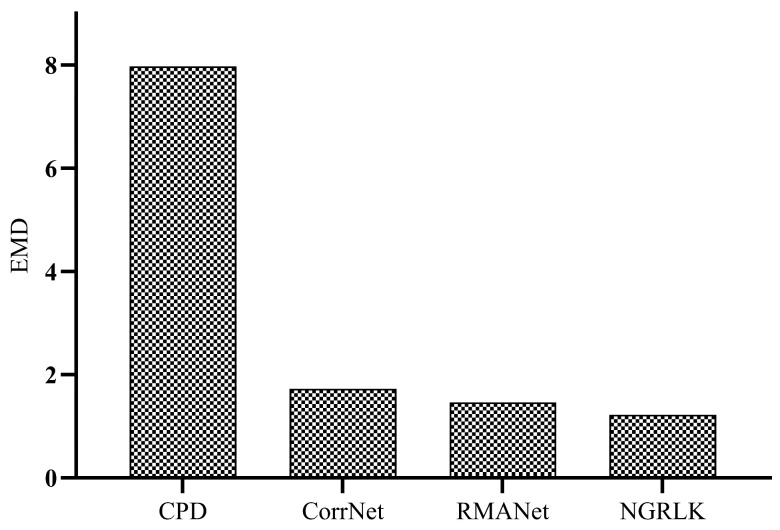
The EMD of CPD (2007) [12], CorrNet3D (2021) [7], RPMNet (2020) [46], and NGRLK.

**Figure 9:**
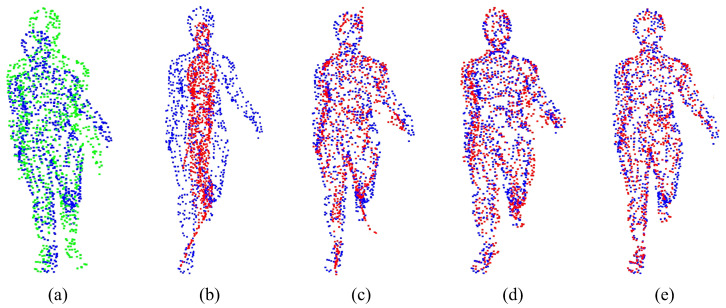
The registration results of CPD (2007) [12], CorrNet3D (2021) [7], RPMNet (2020) [46], and NGRLK on the training dataset. (**a**) The source point cloud and target point cloud (ground truth); (**b**) the registration results of CPD; (**c**) the registration results of CorrNet3D; (**d**) the registration results of RMANet; (**e**) the registration results of NGRLK.

**Figure 10:**
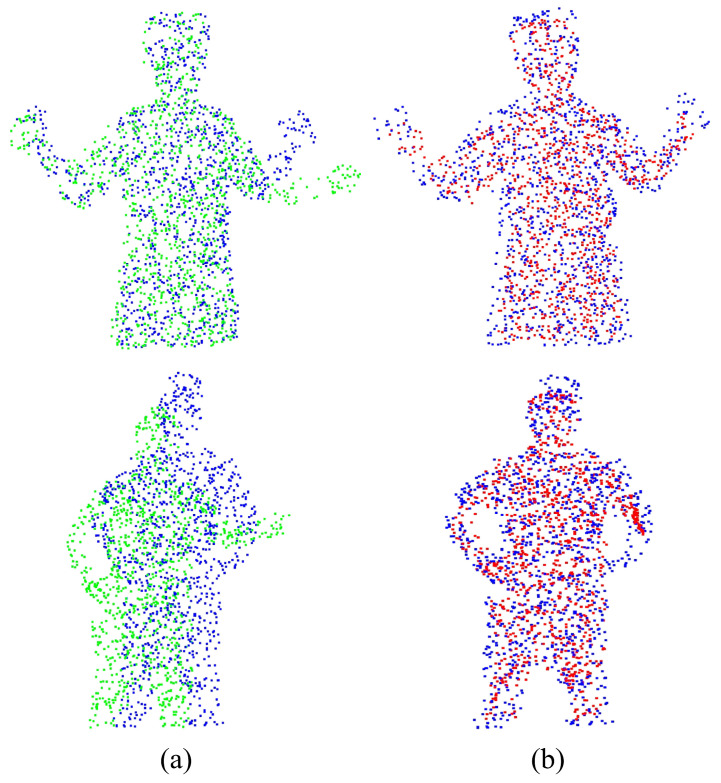
The non-rigid registration results of NGRLK. (**a**) The source point cloud and target point cloud (ground truth); (**b**) the non-rigid registration results of NGRLK on training data.

For the experiment analysis of non-rigid alignment, CPD, F3D, CorrNet3D, RMANet, and Lepard algorithms are selected for comparison. As the CPD and the RMANet do not manifest correspondences, only F3D, CorrNet3D, Lepard, and NGRLK algorithms compute and record the Corr (%) in Figure 6 and Figure 7. The F3D and Lepard mainly perform point cloud feature representation, which can combine with multiple rigid/non-rigid registration algorithms based on point feature correspondences. Only four algorithms, CPD, CorrNet3D, RMANet, and NGRLK, compute the EMD distance in the training dataset Surreal, which are shown in Figure 8. The experimental visual effects of the non-rigid registration on the test dataset SHREC and real sampled data are shown in Figure 9 and Figure 10, respectively.

Figure 6 shows the Corr (%) result of F3D, CorrNet3D, Lepard, and NGRLK algorithms, which indicates that the performance of CorrNet3D, Lepard, and NGRLK is close to each other and significantly better than that of F3D up to 5% error tolerance. When the error tolerance rate is increased to 15%, the CorrNet3D and the NGRLK still show similar performance; the Lepard gradually highlights the advantage of algorithm performance with a 5% higher Corr (%) value. As the error tolerance is increased to 20%, compared with the CorrNet3D, the Lepard outperforms by about 8%, and NGRLK also shows better performance with a 3% higher Corr (%). Throughout the process, the CorrNet3D consistently outperforms the F3D by 5–10% Corr (%). The correspondence percentage can be effectively improved by enhancing the point feature expression capability or adopting more advanced feature-matching algorithms. As the red line indicates the correspondence between the origin and the target point, Figure 7 presents visually that Lepard and NGRLK produce more accurate corresponding points at the same error tolerance. The F3D represents points by a hierarchical feature extraction method (similar to PointNet++ [47]), and both CorrNet3D and NGRLK characterize points by a DGCNN-like approach. The DGCNN is more capable of representing point features and proves that PointNet++ is a subclass feature extraction process, which corresponds to the F3D having the lowest Corr (%) value. Lepard represents point features by synthesizing feature space, position space, and 3D relative distance, which has a strong feature expression capability. More experiment details for the effect of different feature extraction methods on the Corr (%) value are recorded in the subsequent ablation experiments.

The EMD results of the CPD, CorrNet3D, RMANet, and NGRLK non-rigid registration algorithms for the same object are recorded in Figure 8. The CPD has the largest EMD of 0.07937, which is about 6.5 times higher than the other three algorithms. Compared with the advanced non-rigid alignment algorithms based on unsupervised learning in recent years, the EMD 0.01219 of NRGLK is lower than the EMD 0.01728 of CorrNet3D and EMD 0.01462 of RMANet. The NGRLK proposed in this paper has the smallest registration error. Figure 9 shows the visual registration results of the CPD, CorrNet3D, RMANet, and NGRLK, where the blue represents the target object, the green is the original object and the red shows the registrated object with different methods. From the visual perception, the NGRLK, CorrNet3D, and RMANet can perform non-rigid registration. When dealing with a larger degree of non-rigid deformation, the CPD registration performance decreases, and the NGRLK has the smallest alignment error.

Figure 10 exhibits the alignment results of NGRLK on real data to validate the availability of the algorithm, where Figure 10a denotes the source-target point cloud of self-collected half-body point cloud data, and Figure 10b denotes the non-rigid alignment results. The first set of self-sampled half-body point cloud data has noise and a small range of deformation, and the second set of self-sampled full-body point cloud data has noise, rotational bias, and large deformation. The registration results validate that the NGRLK can perform non-rigid registration on real sampled non-rigid data with good generalization ability and usability.

### 5.4. Rigid Experiment

**Figure 11:**
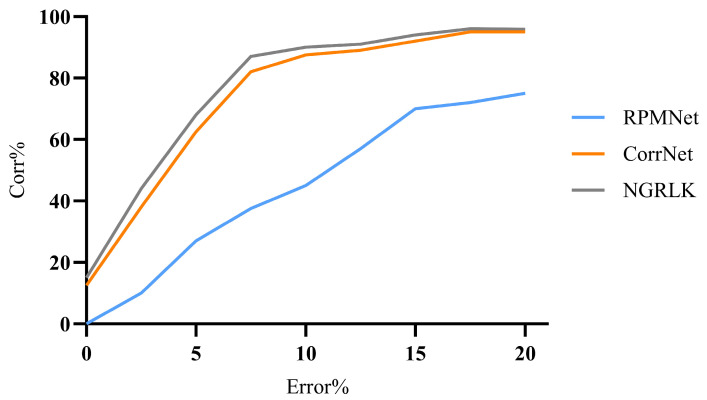
The Corr% of RPMNet (2020) [46], CorrNet3D (2021) [7], and NGRLK.

**Figure 12:**
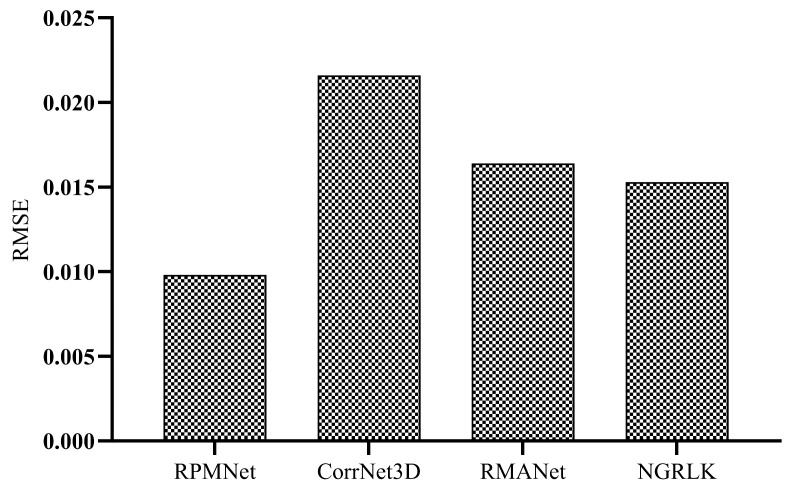
The RMSE of RPMNet (2020) [46], CorrNet3D (2021) [7], RMANet (2021) [6], and NGRLK.

**Figure 13:**
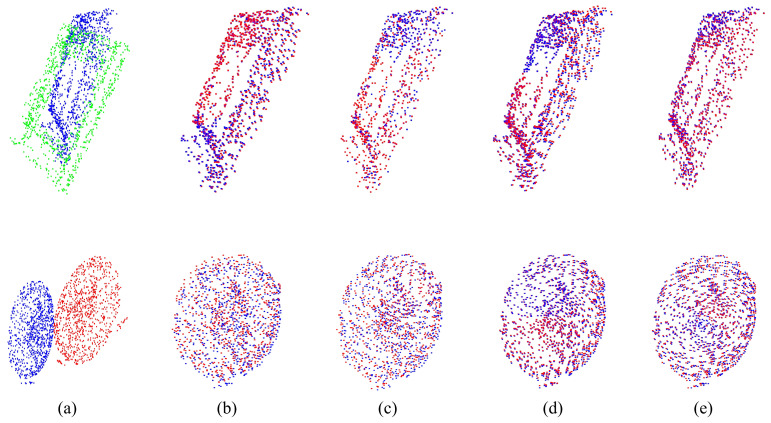
The registration results of RPMNet (2020) [46], CorrNet3D (2021) [7], RMANet (2021) [6], and NGRLK. (**a**) The source point cloud and target point cloud (ground truth); (**b**) the registration results of RPMNet; (**c**) the registration results of CorrNet3D; (**d**) the registration results of RMANet; (**e**) the registration results of NGRLK.

For the rigid registration experiment analysis, the RPMNet, CorrNet3D, and RMANet algorithms were selected for comparison. The RPMNet, CorrNet3D, and NGRLK (RMANet does not reflect the correspondence) performed the Corr (%) computation, which is recorded in Figure 11. The registration performance of the RPMNet, CorrNet3D, RMANet, and NGRLK is compared on the ModelNet40 dataset, and the RMSE value is recorded in Figure 11. Moreover, the alignment results of the four registration models on ModelNet40 and real sampled data are shown in Figure 12 and Figure 13, respectively.

Figure 11 records the rigid registration Corr (%) of the RPMNet, CorrNet3D, and NGRLK and indicates good performance. When the error tolerance exceeds 7.5%, the Corr (%) of both CorrNet3D and NGRLK is above 80%, and the Corr (%) of RPMNet is only 40%. While the error tolerance is increased to 15%, the Corr (%) of all three algorithms exceeds 70%, and that of both CorrNet3D and NGRLK in particular exceeds 90%. The NGRLK has the highest Corr (%) at any error tolerance and presents the best performance.

Figure 12 records the numerical analysis of the registration errors for RPMNet, CorrNet3D, RMANet, and NGRLK. As the representative of the rigid registration algorithm, RPMNet has the smallest RMSE of 0.0098. Among CorrNet3D, RMANet, and NGRLK, NGRLK has the smallest RMSE of 0.0153, which is about 56% higher than that of RPMNet. CorrNet3D and RMANet have larger RMSEs of 0.0216 and 0.0164, which are about 2.2 and 1.67 times higher than that of RPMNet, respectively.

**Figure 14:**
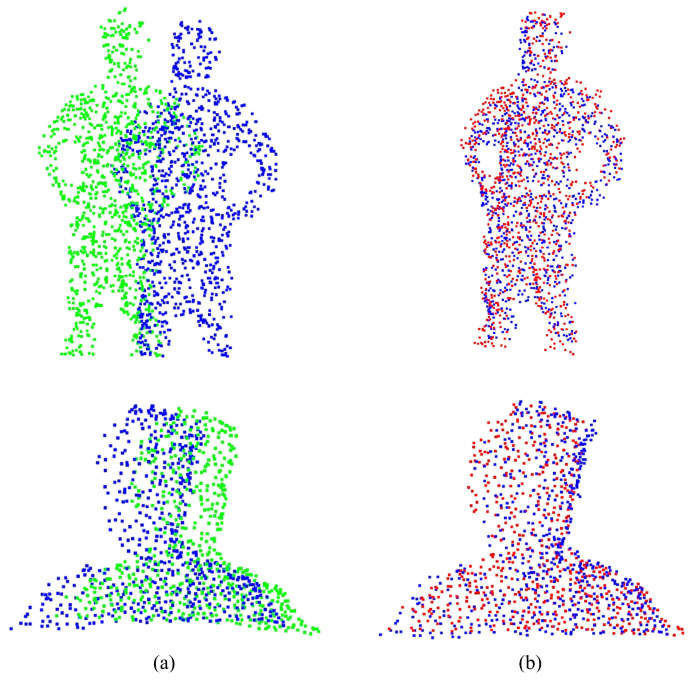
The registration results of NGRLK. (**a**) The source point cloud and target point cloud (ground truth); (**b**) the registration results of NGRLK on real sampled data.

Figure 13 demonstrates the rigid registration results of RPMNet, CorrNet3D, RMANet, and NGRLK on the ModelNet40 noisy dataset, which is generated in the same way as our previous research DIRGMR [41]. The intuitive visual comparison shows that all four algorithms can complete the rigid registration on noisy data well. To further validate the usability of the registration model proposed in this paper, Figure 14 summarizes the rigid registration results of the NGRLK on platform-sampled data. The real sampled data are noisy and occluded, and the visual registration verifies that the NGRLK algorithm can complete rigid alignment on the noisy data well.

### 5.5. Ablation Experiment

**Figure 15:**
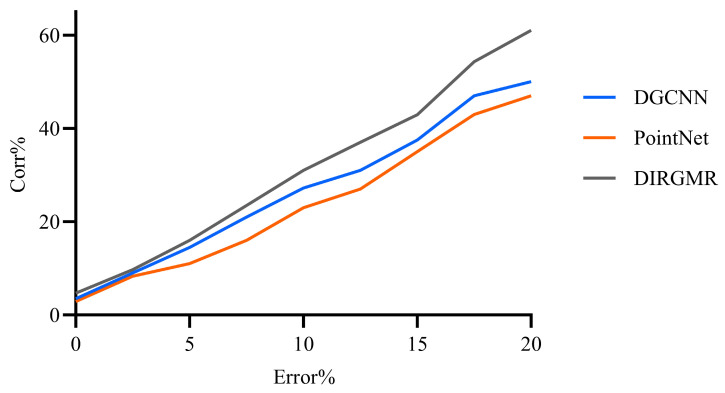
The impact of DGCNN (2019) [40], PointNet (2017) [27], and DIRGMR (2024) [41] feature extraction on Corr (%).

In this subsection, we perform sufficient ablation tests on the loss function of $L_{surf}$, $L_{space}$, and $L_{CD}$ in NGRLK with different feature extraction modules. By default, the pluggable feature extraction module in NGRLK adopts DGCNN (2019) [40], which is compared to the feature description method adapted with PointNet (2017) [27] and the feature extraction module proposed with non-regular shape factors in DIRGMR (2024) [41], respectively. With other experiment conditions unchanged, Figure 15 records the registration Corr (%) corresponding to the different feature extraction methods. The analysis points out that when the error tolerance is less than 2.5%, the NGRLK is almost unaffected by the expression ability of the point features, and the registration Corr (%) is relatively low (less than 10%). While the error tolerance is increased to 5%, the registration Corr (%) is affected by the feature extraction method. In particular, when the error tolerance rises to 10%, the intuitive visualization shows that the default configuration of the registration Corr (%) improves by about 5% over the NRGLK main framework adapted to the PointNet feature extraction approach. As the error tolerance continues increasing to 20%, the Corr (%) of the NGRLK fused with DIRGMR is consistently higher than that of the default configured NGRLK and the NGRLK main framework adapted to PointNet, with a maximum difference of about 12%. The feature representation fused with the irregular shape factor can effectively improve the accuracy of the registration Corr (%). At the same error tolerance, the registration Corr (%) of the NGRLK with default configuration is also consistently higher than that fused with PointNet by between 3 and 5%, which is presumably because PointNet is not as expressive as DGCNN for point feature extraction.

**Figure 16:**
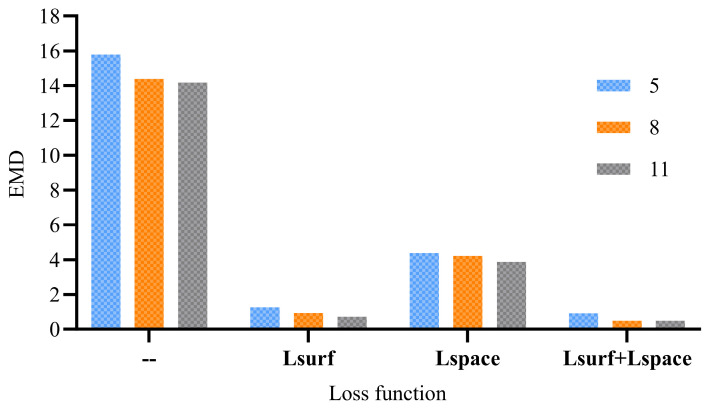
The loss function effect on registration accuracy.

Then, tests are mainly conducted to analyze the effect of different loss functions on EMD. Specifically, the effects of $L_{surf}$ and $L_{space}$ on the accuracy of the registration results are compared at different loop stages. When the number of cycles is set to 5, 8, and 11, Table 1 records the registration EMD corresponding to $L_{surf}{\;+\;L}_{surf}$, missing both $L_{surf}{\;+\;L}_{surf}$, and missing either $L_{surf}$ or $L_{space}$. Figure 16 compares the effect of loss functions on the accuracy of the registration results through histograms.

Combining Table 1 and Figure 16, the analysis reveals that the EMD decreases from 15.79 to 14.37 when the number of cycles increases from 5 to 8, without $L_{surf}$ and $L_{space}$. When the number of cycles continuously increases to 11, the EMD only decreases by 0.21. When different loss functions are used, the analysis points out that the EMD values change with a similar pattern. For the records of using $L_{surf}$ or $L_{space}$ alone, the analysis validates that the EMD values change with a similar pattern, which is the small decrease in EMD when the number of loops increases from 5 to 8, and the lower decrease in EMD when the number of loops increases to 11. The above tests illustrate the limited improvement in model training accuracy by the number of loops without adapting an effective loss function. However, when the number of cycles is fixed, both $L_{surf}$ and $L_{space}$ can improve the registration accuracy substantially (EMD values decrease by a factor of 3–30), where $L_{surf}$ having a greater impact than $L_{space}$ for $L_{surf}$ imposes constraints on deformations directly, whereas $L_{space}$ imposes constraints on the relative position of the points in local space that remain unchanged after a single rigid change. In addition, the superposition of $L_{surf}$ and $L_{space}$ can reduce the EMD by nearly 0.7 times, which also indicates that the designed loss function can effectively constrain the learning direction of the NGRLK parameters.

## 6. Conclusions

Here, we propose an unsupervised non-rigid registration model of dual recurrent computation. Through elastic fine-tuning incremental transformations, a more detailed rigid transformation can be obtained. Combining the attention mechanism and recurrent neural network, the weight of each rigid transformation is updated, and the influence of invalid transformations can be effectively eliminated. In the unsupervised learning framework, a two-dimensional manifold representation is utilized for both the dimensional reduction and reconstruction of point cloud data, complemented by a specifically designed loss function that enforces deformation similarity constraints to guide model training. Moreover, the spatial distribution loss function is designed to make the rigid transformation as rigid as possible. Compared with representative advanced registration models in recent years, NGRLK achieves state-of-the-art performance with 0.01219 earth mover’s distance (EMD) for non-rigid registration and 0.0153 root mean square error (RMSE) for rigid alignment, outperforming approaches it has been compared to by averages of 15% and 50%, respectively, on standard benchmarks. Currently, the proposed NGRLK conducts the experimental scene mainly for indoor object-level and asynchronous interactive scenarios. In the future, research works should explore outdoor and real-time registration performance to expand the application.

## Figures and Tables

**Table 1:** The registration accuracy comparison results of $L_{surf}$ and $L_{space}$.

Loss	Recurrent Number	EMD
-	5	15.79
-	8	14.37
-	11	14.16
$L_{surf}$	5	1.26
$L_{space}$	5	4.37
$L_{surf}+L_{space}$	5	0.924
$L_{surf}$	8	0.943
$L_{space}$	8	4.21
$L_{surf}+L_{space}$	8	0.505
$L_{surf}$	11	0.732
$L_{space}$	11	3.879
$L_{surf}+L_{space}$	11	0.503

## Data Availability

The original contributions presented in this study are included in the article.
